# Common sources of linguistic conflict engage domain-general conflict control mechanisms during language comprehension

**DOI:** 10.3758/s13415-025-01267-3

**Published:** 2025-02-12

**Authors:** Megan A. Boudewyn, Yaqi Xu, Ashley R. Rosenfeld, Nathan P. Caines

**Affiliations:** University of California, Santa Cruz, CA USA

**Keywords:** Language comprehension, Electroencephalograms, Cognitive control, Theta

## Abstract

The current study tested the hypothesis that lexical ambiguity, a common source of representational conflict during language comprehension, engages domain-general cognitive control processes that are reflected by theta-band oscillations in scalp-recorded electroencephalograms (EEG). In Experiment 1, we examined the neural signature elicited by lexically ambiguous compared to unambiguous words during sentence comprehension. The results showed that midfrontal theta activity was increased in response to linguistic conflict (lexical ambiguity). In Experiment 2, we examined postconflict adaptation effects by comparing temporarily ambiguous sentences that followed previous instances of conflict (other temporarily ambiguous sentences) to those that followed a previous low-conflict (unambiguous) sentence. A midfrontal theta effect associated with linguistic conflict was again found in Experiment 2, such that theta was increased for temporarily ambiguous sentences that followed previous low-conflict (unambiguous) sentences compared with those that followed previous high-conflict (temporarily ambiguous) sentences. In both experiments, facilitated lexical semantic processing was also observed for words that came after the point of conflict, which may reflect a downstream “benefit” of cognitive control engagement. Overall, our results provide novel insights into the neurocognitive mechanisms underlying conflict processing in language comprehension and suggest that the same neural computations are involved in processing nonlinguistic and linguistic conflict.

**Supplementary Information:**

The online version contains supplementary material available at 10.3758/s13415-025-01267-3.

## Introduction

Language is rife with conflict. For example, readers and listeners frequently encounter lexically ambiguous words, meaning words that are compatible with more than one interpretation (e.g. *ball*, which can refer to an *object with which you can play a game* or to a *formal dance party*). Lexical ambiguity often results in the brief simultaneous activation of both meanings (Altarriba & Gianico, 2003; Simpson, 1981, 1984; Van Petten, 2002; Van Petten & Kutas, 1987, 1988). It therefore can be understood as a source of representational conflict, as encountering a lexically ambiguous word can lead to the activation of multiple competing representations that are in conflict with one another. Although the lexically ambiguous word may activate multiple mental representations of meaning, typically only one meaning is intended and is the best fit for the context. Decades of language processing research have shown that readers and listeners can use a variety of sources of information to help resolve this conflict, including previous context and the overall frequency of the possible meanings of the lexically ambiguous word (see Rodd, 2018 for a recent review). However, while a great deal is known about the types of information that can influence how readers and listeners ultimately interpret the meaning of the word, less is known about the underlying conflict detection and resolution mechanisms that are involved.

Recent work has proposed that domain general cognitive control mechanisms are engaged to resolve representational conflict during language comprehension (Ness et al., 2023). Cognitive control refers to a set of mechanisms that enable flexible, adaptive cognitive processing based on current goals and context. Conflict monitoring accounts of cognitive control propose a conflict detection mechanism that is sensitive to the presence of simultaneously active (conflicting) response representations (Botvinick et al., 2001, 2004). The detection of conflict is thought to serve as a signal to increase top-down cognitive control to guide responding according to the current goals of the task. Increased top-down control is hypothesized to take the form of a biasing mechanism that enhances the activation of the representation that is most compatible with current goal(s) or context (Botvinick et al., 2001, 2004; Van Veen & Carter, 2006). Many cognitive control tasks are designed to elicit a conflict between a default response of some sort (e.g., word naming in the Stroop task) and an alternative response that is specific to the task (e.g., color naming in the Stroop task). These conditions generate a conflict between the default and task-appropriate responses, and cognitive control is thought to be engaged to enhance activation of the task-appropriate response and ultimately promote task-appropriate responding.

Building upon this conceptualization of cognitive control, Ness et al. (2023) proposed that the same biasing mechanism that is at work in guiding task-appropriate responding in cognitive control tasks is also engaged by linguistic conflict (i.e., the presence of multiple competing linguistic representations) and serves to boost the most likely of all active linguistic representations (see also January et al., 2009; Novick et al., 2005). The biasing mechanism proposed by Ness et al. (2023) operates via reciprocal excitatory (activation enhancing) connections between the biasing mechanism and activated linguistic knowledge representations. Once engaged by the detection of linguistic conflict, the biasing mechanism boosts the activation levels of the conflicting linguistic knowledge representations in proportion to their existing levels of activation, which serves to further increase the activation level of the “leading” representation in an iterative process that ultimately leads to the resolution of the conflict in favor of the most likely linguistic representation. Importantly, Ness et al. (2023) propose that the biasing mechanism is connected to linguistic knowledge representations, with these activation levels being influenced by factors such as previous context and frequency of occurrence (which, as noted above, are known to guide the interpretation of lexically ambiguous words). In this way, the biasing mechanism has access to the information needed to determine which representation is most likely and should be enhanced. Ness et al. (2023) do not propose direct links between their model and underlying neurocognitive mechanisms, noting that it is has not yet been established that cognitive control engagement during language comprehension elicits a neural response akin to that observed in non-linguistic cognitive control tasks. Ness et al. (2023) do note, however, that theta oscillations may be a candidate neural mechanism that supports conflict control processing during language comprehension, but also note that this has not yet been empirically demonstrated.

One of the key goals of the current study was to test the hypothesis that oscillatory theta activity, a signal derived from scalp-recorded EEG, reflects the neural mechanisms involved in conflict control processing during language comprehension. EEG activity in the theta frequency band (~4–8 Hz) is widely considered to be a cognitive control signal, particularly when recorded from mid-frontal electrodes (Cavanagh et al., 2012; Cavanagh & Frank, 2014; Eisma et al., 2021). Theta band activity has been observed at mid-frontal electrodes in response to conflict on traditional cognitive control tasks, such as the Stroop task (Boudewyn & Carter, 2018; Eschmann et al., 2018; Oehrn et al., 2014), Simon task (Cavanagh et al., 2012; Cohen & Donner, 2013), and Flanker task (Cavanagh et al., 2009; Nigbur et al., 2012). Mid-frontal theta is thought to be generated by the anterior cingulate cortex (ACC), which likely supports the initial detection of conflict across multiple tasks and domains. Once conflict is detected, the ACC is thought to engage lateral prefrontal cortex (PFC), either directly (Botvinick et al., 2001, 2004; Kerns et al., 2004) or indirectly via the locus coeruleus (Botvinick et al., 2001; Verguts & Notebaert, 2009), to facilitate the resolution of the competition between the conflicting representations.

Some data suggest that conflict during speech production also engages these domain general conflict control circuits and neural computations. For example, increased theta activity has been observed in response to high-conflict Stroop-like conditions during spoken word production (e.g., saying the word *dog* while being presented with a picture of a cat) (Piai et al., 2014; Shitova et al., 2017). Studies of language production have also found event-related potential (ERP) evidence for shared neural computations in cases of linguistic conflict and nonlinguistic conflict (Riès et al., 2011, 2021). Specifically, the error-related negativity (ERN) is an ERP component derived from EEG recordings that shares a time course and scalp distribution with the mid-frontal conflict-related theta described above. The ERN has been consistently observed to be larger when time-locked to errors than to correct responses in a variety of tasks (Falkenstein et al., 1991; Gehring et al., 1993, 2012), although it is also present for correct responses under conditions of high response conflict, suggesting that it is a marker of conflict detection rather than of the commission of an error per se (Carter et al., 1998; Cavanagh & Frank, 2014; Vidal et al., 2000; Yeung et al., 2004). In a set of studies, Ries & colleagues showed that the ERN can be elicited during speech production, which is thought to require cognitive control and specifically conflict monitoring and detection to avoid speech errors (Riès et al., 2011, 2021). Based on these findings, the same neural computations proposed to be at work in nonlinguistic conflict processing (and reflected by mid-frontal theta activity and the ERN) have been hypothesized to support linguistic conflict monitoring during speech production (Nozari et al., 2011; Nozari & Novick, 2017). However, it is an open question whether general neural computations associated with conflict monitoring and detection support linguistic conflict resolution during comprehension. The primary goal of the current study was to address this question.

### The current study

The current study consisted of a set of two experiments. In Experiment 1, EEG was recorded while participants read sentences containing a lexically ambiguous word, and sentences that were fully unambiguous. Many lexically ambiguous words have a dominant sense (e.g., the *object with which you can play a game* meaning of *ball*), which might be considered the default meaning of the word, and a subordinate sense (e.g., the *formal dance party* meaning of *ball*). Participants read sentences that were initially compatible with either sense of a lexically ambiguous word, which would be expected to activate either only the dominant meaning of the word, or potentially both the dominant and subordinate meanings of the word (Simpson, 1981; Tabossi et al., 1987). In either case, the sentences continued on to evoke conflict between dominant and subordinate representations of the word, as a later word in each sentence revealed that the subordinate meaning was intended. For example, in the sentence “The ball was by invitation only,” the beginning portion of the sentence (“*The ball was by*…”) is compatible with both the dominant and subordinate senses of the word. When the reader encounters “…invitation” (disambiguation word), it becomes clear that the most likely interpretation of the ambiguous word is the subordinate meaning, which should lead to the simultaneous activation of two conflicting representations (one consistent with the dominant meaning, and the other consistent with the subordinate meaning).

We expected to find increased theta activity at the disambiguation word in the ambiguous compared to unambiguous condition, to the extent that conflicting linguistic representations during comprehension engage the same cognitive control mechanisms as nonlinguistic conflict. As noted, the disambiguation word is the point at which participants first encountered information inconsistent with the dominant meaning of the ambiguous word and, thus, is the point at which we hypothesized that conflict control processes would be maximally engaged. We also examined the N400 response to the post-disambiguation word to examine lexical semantic processing after readers encountered linguistic conflict. The N400 is a negative-going ERP that reflects the relative ease of lexical-semantic processing (Swaab et al., 2012). Previous work has found reduced N400 amplitudes for words in unambiguous sentences compared to words in the post-disambiguation region of ambiguous sentences, indicative of facilitated lexical-semantic processing for words in the unambiguous (no-conflict) condition (Hagoort & Brown, 2015).

**Fig. 1:**
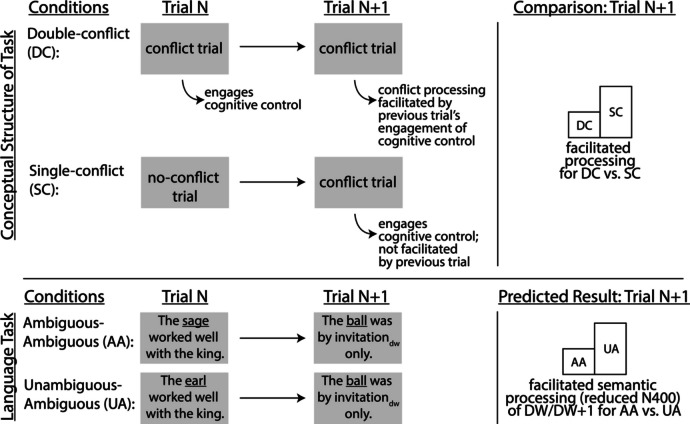
Experiment 2 design overview. Sentences are shown in their entirety for ease of reading; in the actual experiment, words were presented one at a time in rapid serial visual presentation (RSVP) format. Also, lexically ambiguous words are underlined and disambiguation words are denoted with a “dw” subscript for visualization purposes only; in the actual experiment they were not emphasized in any way

In Experiment 2, we looked for trial-to-trial adaptive control effects in the theta response to conflict arising from lexical ambiguity during comprehension. Trial-to-trial adaptive control effects, which are sometimes also called congruence sequence effects, refer to a well-studied phenomenon in conflict-based cognitive control tasks (e.g., the Stroop) in which the second of two consecutive high-conflict trials tends to processed more quickly and accurately than a low-conflict trial that follows a high-conflict trial (Gratton et al., 1992; Ullsperger et al., 2005). This effect has been interpreted as evidence for sustained cognitive control, such that a conflict monitoring mechanism is engaged by the first high-conflict trial, leading to enhanced top-down cognitive control, which then carries over and benefits the subsequent high-conflict trial. In order to examine potential trial-to-trial adaptive control effects during language comprehension, we examined the processing of ambiguous sentences that immediately followed other ambiguous sentences compared with those that followed unambiguous sentences (see Fig. 1 for design overview). Specifically, we tested for the presence of a theta effect at the disambiguation word by comparing ambiguous sentences that followed unambiguous sentences (Unambiguous-Ambiguous condition) to those that followed other ambiguous sentences (Ambiguous-Ambiguous condition). If present, this effect would provide evidence for postconflict trial-to-trial facilitation during language comprehension. As in Experiment 1, we also examined downstream effects on lexical semantic processing after the disambiguation word, as the sentences continued to unfold. To the extent that adaptive control in the AA condition leads to facilitated lexical semantic processing, we expected to find reduced N400 amplitudes for AA compared to UA trials at the post-disambiguation word.

## Experiment 1

As described, the goal of Experiment 1 was to compare the theta response at the disambiguation word and subsequent semantic processing in temporarily ambiguous to unambiguous sentences.

### Experiment 1: methods

#### Participants

This experiment received research ethics committee approval from the UC Santa Cruz institutional review board. We used the *mlmpower* package (Enders et al., 2023) in *R* (R Core Team, 2013) to determine that a sample size of approximately 30 participants and 52 trials per condition would enable us to detect a medium effect size (*R*^*2*^ = .13) for fixed effects using the multilevel modeling approach described below, based on α = 0.05 and (1-β) = 0.85. Thirty participants were recruited from the University of California, Santa Cruz (20 females; 9 males; 0 nonbinary; 1 did not report; average age 19 [range 18–22] years). All participants were right-handed native speakers of English.

#### Task

The task was presented using Neurobehavioral Systems Presentation (www.neurobs.com). Stimuli consisted of sentences presented visually, one word at a time in rapid serial visual presentation (RSVP) format (300-ms duration per word, 200-ms interstimulus interval). Experimental sentences contained an ambiguous word (e.g., *ball*) or unambiguous word (e.g., *party*) but were otherwise identical. Ambiguous words were selected that had a clear dominant sense (e.g., the *object with which you can play a game* sense of the word *ball*) and a subordinate sense (e.g., the *formal dance party* sense of the word *ball*), based on an independent sample of 100 participants who were asked to type in the first word that came to mind that they found to be related in meaning to the prompt (data collected online using Psytoolkit). On average, the independent sample responded with words that were related to the dominant meaning of the words used in this task 86.6% of the time (range 61.75–100%) and to the subordinate meaning 8.32% of the time (range 0–29.5%).

Sentences were constructed such that they were compatible with both senses of the ambiguous word up until a disambiguation word, at which point the sentence context biased in favor of the subordinate sense of the ambiguous word (e.g., *The ball/dance was by invitation only*; “invitation” is the disambiguation word in this example). The goal of this manipulation was to create maximal conflict at the disambiguation word in the ambiguous condition (whereas the unambiguous condition would not lead to such conflict). Specifically, in the ambiguous condition, there is a conflict between the previously assumed meaning of the developing sentence (compatible with the dominant sense) and the meaning once the disambiguation word and further context becomes available (compatible with the subordinate sense).

A total of 105 experiment sentence sets were created. Two counterbalanced lists were created such that all items appeared in each condition across lists, but within a list no items were repeated. Each participant was presented with one list. Thus, each participant read 52 to 53 unique items per condition. Thirty filler sentences containing no ambiguous words were also included. Comprehension questions were included to encourage attentive reading; questions only appeared after filler sentences (average accuracy 98.5%; range 89–100%).

#### EEG recording and preprocessing

EEG was recorded by using a Brain Products active electrode ActiCHamp system with 32 scalp electrodes and 2 sets of passive bipolar EOG electrodes (to record vertical EOG: 1 below the left eye and 1 above the left eye; to record horizontal EOG: 1 on the left outer canthus and 1 on the right outer canthus). Channels were referenced online to electrode TP9 (proxy for left mastoid). Data were re-referenced offline to the average of TP9/TP10. Data were recorded with a sampling rate of 1000 Hz and electrode impedances were kept below 10 kOhms.

EEG data was preprocessed offline using Matlab with EEGlab Toolbox (Delorme & Makeig, 2004) and the ERPlab plugin (Lopez-Calderon & Luck, 2014). Our preprocessing approach is detailed in Boudewyn et al., 2023 and summarized here. The data were high-pass filtered using a noncausal Butterworth filter (half-amplitude cutoff of 0.05 Hz, 12 dB/octave slope) and screened for nonfunctioning channels. Nonfunctioning channels were defined as channels with unusable data during one-third or more of the total recording time. Independent component analysis (ICA) was used to correct for eye blinks and horizontal eye movements by using the approach described in Boudewyn et al. (2023). The nonfunctioning channels were excluded from ICA and then interpolated after ICA using a spherical spline algorithm. The continuous EEG was then segmented into epochs of −200 to 1500 ms (for ERP analysis) and −2000 to 2000 ms (for time-frequency analysis), time-locked to disambiguation word onset. Epochs were then screened for any remaining artifacts, using ERPlab functions for detecting extreme values (default set to −150 to 150 uV). On average, 16.98% of trials were rejected owing to the presence of artifacts, with an average of 43 trials per condition remaining in each dataset (range 24–53).

#### Data analysis

Two key measures were extracted from the data: theta power time-locked to the disambiguation word and N400 amplitude in response to the post-disambiguation word.

##### Theta-power

Single-trial estimates of theta power were calculated using the EEGlab toolbox, by convolving single-trial epochs with three-cycle complex Morlet wavelets. Baseline-corrected power estimates (baseline window: −400 to 50 ms) corresponding to average theta power (5–8 ms) in the 200–400-ms window were calculated for each trial.

##### N400 amplitude

The mean amplitude corresponding to the N400 time window (300–500 ms) following the onset of the post-disambiguation word was calculated (baseline window: −200 to 0 ms).

### Experiment 1: results

All statistical analyses were performed in *R* (R Core Team, 2013) using the *lme4* package (Bates et al., 2014). Significant effects of condition are reported below in the main text. Significant main effects of scalp distribution are not reported in the main text, because these are not interpretable other than to indicate that overall voltage values varied across electrodes. However, significant interactions of scalp distribution variables and condition are interpretable and are reported in the main text below. Please see supplemental materials for full model output.

#### Conflict-related theta activity at disambiguation word

**Fig. 2:**
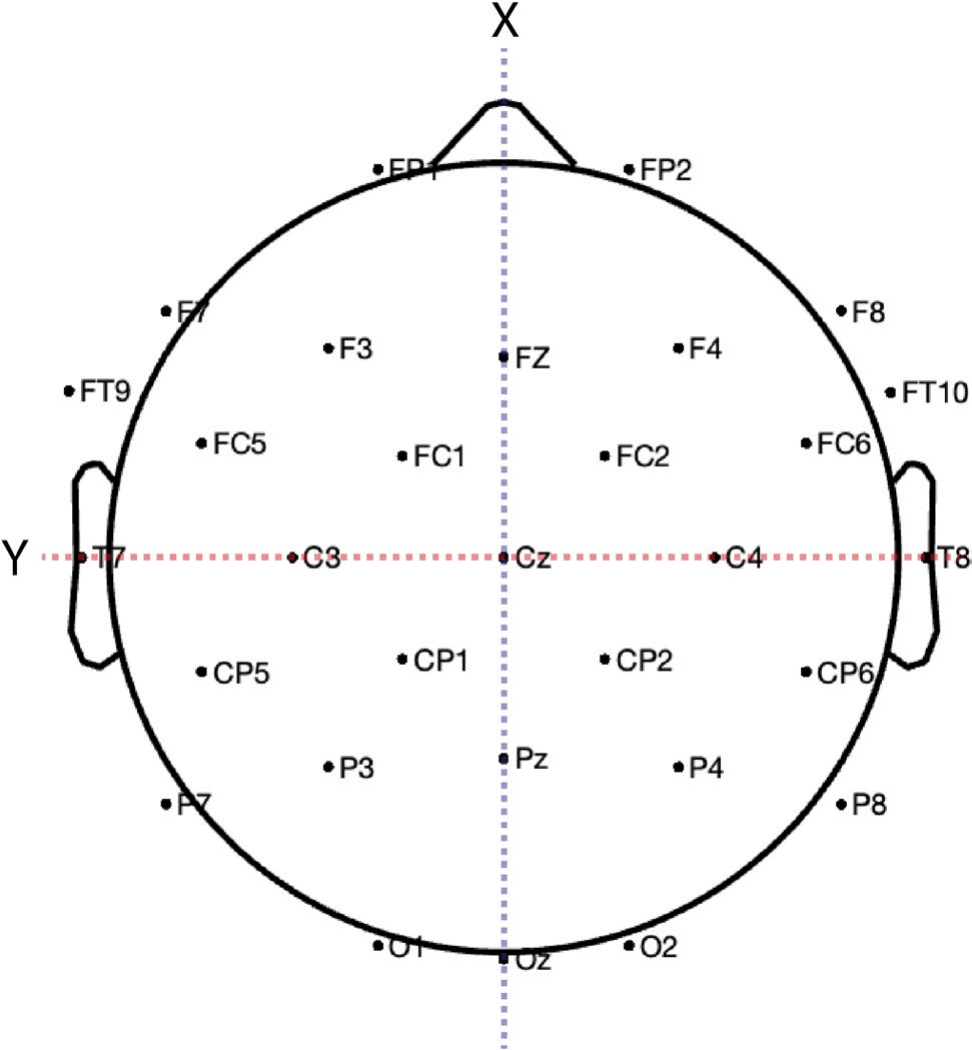
Electrode channel layout. Scalp distribution coordinate system displayed with dashed lines. The X-axis (dashed blue) captures the anterior-to-posterior plane, passing through the nasion. The Y-axis (dashed red) captures the left-to-right plane, passing through the pre-auricular points. The Z-axis (not pictured) captures the superior-to-inferior plane, passing through the vertex (Cz)

We first assessed the extent to which condition predicted theta-activity in response to the disambiguation word on a trial-by-trial basis. We used a linear mixed effects regression model with fixed effects of condition (Unambiguous, Ambiguous) and the X, Y, and Z coordinates of each electrode (Boudewyn et al., 2024; Winsler et al., 2018, 2023). Specifically, an electrode’s X-dimension value captured its position along the anterior-to posterior axis, whereas the Y-dimension captured the left-to-right axis and the Z-dimension captured the superior-to-inferior axis corresponding to electrode location (Fig. 2). Condition was treatment-coded by using Unambiguous as the reference condition. The model intercept therefore reflects the reference level of condition (Unambiguous). Because we did not expect potential EEG effects would necessarily be present or equivalent at all electrode locations, we also included interaction terms between X-Y-Z coordinates and condition to capture the scalp distribution of any observed effects of condition. For example, an interaction between condition and the X-distribution predictor would capture a difference between conditions that was maximal at anterior electrode sites. The random effects structure for the model included random intercepts for participants and items, and by-participant random slopes for the effect of condition.

**Fig. 3:**
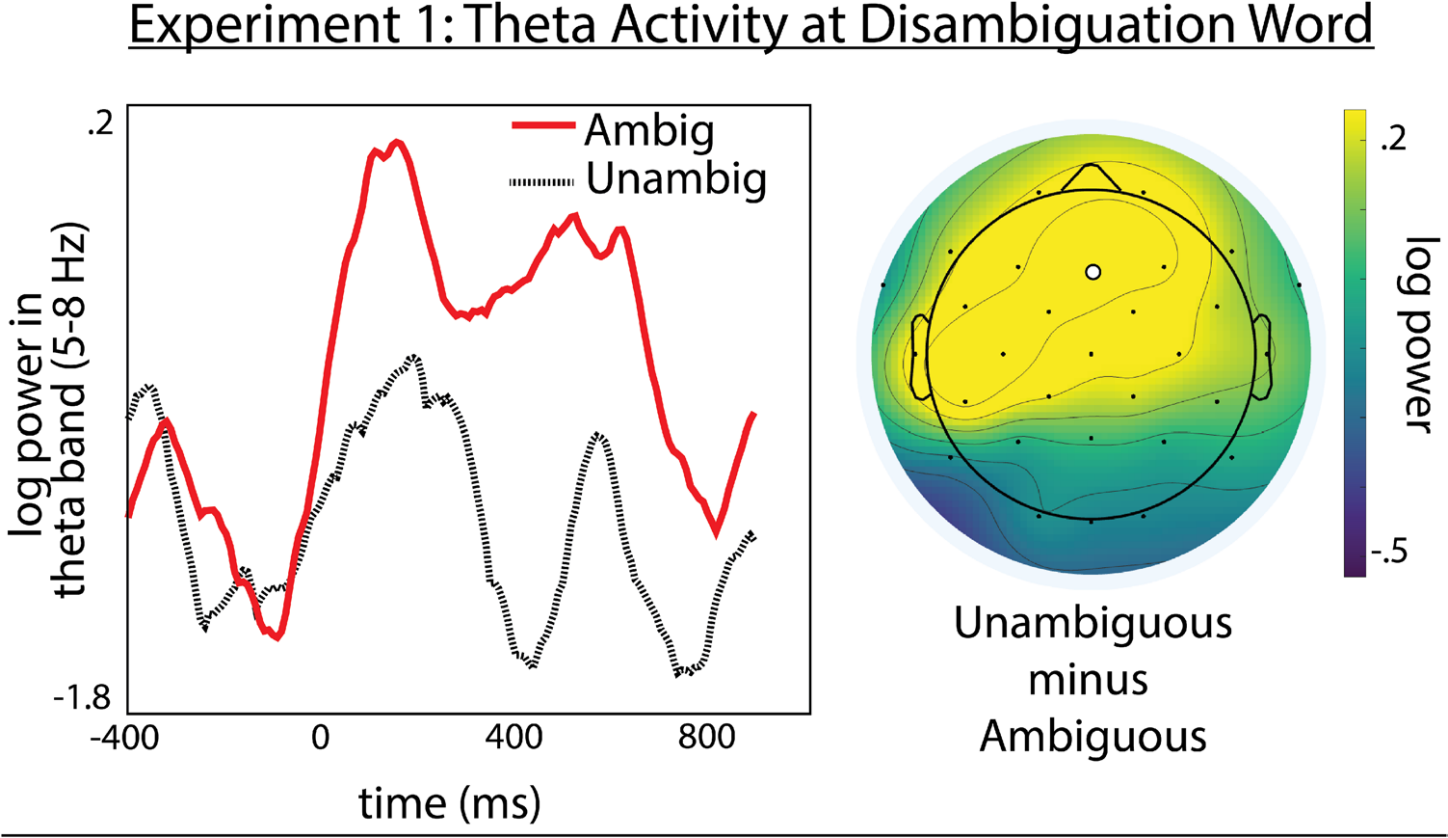
Conflict-related theta activity at disambiguation word in experiment 1. Left: Theta (5–8 Hz) power time-course time-locked to the disambiguation word at electrode Fz. Right: topographic map showing distribution of theta power for Ambiguous minus Unambiguous condition. Electrode Fz denoted by a white circle

The results showed significant interactions between condition and the X-distribution (*β* = −3.304e-04, *SE* = 1.297e-04, *p* = 0.0109) and Z-distribution predictor (*β* = −4.262e-04, *SE* = 1.813e-04, *p* = 0.0187) variables. This interaction reflects a significant increase in theta power for the Ambiguous condition compared with the Unambiguous condition at mid-frontal electrode sites. Results are summarized in Fig. 3.

#### Downstream effects on lexical semantic processing

We next assessed the extent to which condition predicted the N400 amplitude of the post-disambiguation word on a trial-by-trial basis, using the same approach we adopted to assess conflict-related theta activity at the disambiguation word. We fit a linear mixed-effects regression model that included fixed effects of condition (Unambiguous, Ambiguous) and scalp-distribution variables corresponding to X (front-to-back), Y (left-to-right), and Z (superior to inferior) coordinates for each electrode (Fig. 2), as well as interaction terms between condition and each of the X-Y-Z distribution predictors to capture the scalp distribution of any observed effects of condition. As above, condition was treatment-coded using Unambiguous as the reference condition. The model intercept therefore reflects the reference level of condition (Unambiguous). The random effects structure for the model included random intercepts for participants and items, and by-participant random slopes for the effect of condition.

**Fig. 4:**
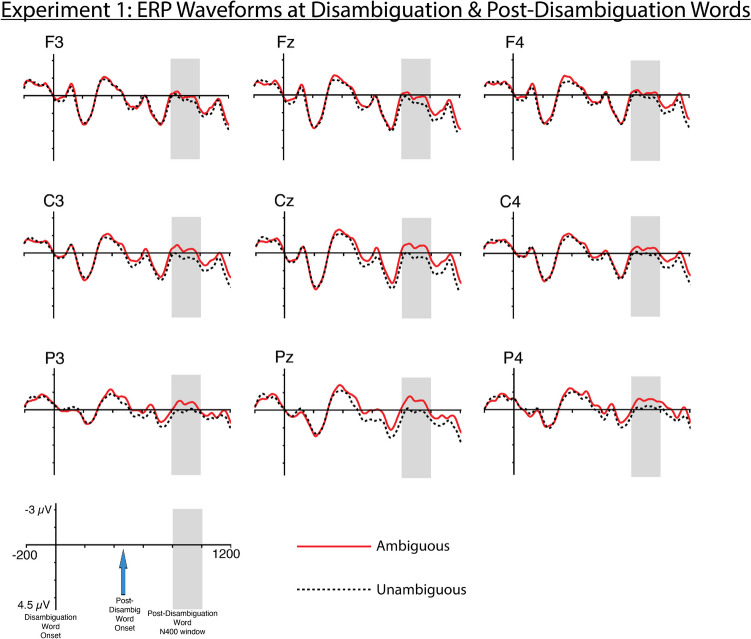
Experiment 1 ERP waveforms at the disambiguation and post-disambiguation words. Waveforms were time-locked to the disambiguation word to provide a visualization of ERP amplitudes throughout the disambiguation region. The post-disambiguation word onset 500 ms after the disambiguation word. Analysis focused on the N400 to the post-disambiguation word (shown in gray shading). Negative is plotted up

The results showed a significant interaction between condition and the Z-distribution predictor (*β* = −5.181e-03 ; *SE* = 2.482e-03; *p* = 0.0369). This interaction reflects the significantly greater N400 amplitudes for post-disambiguation words in the Ambiguous than Unambiguous conditions at central electrode sites (Fig. 4).

## Experiment 2

Experiment 1 demonstrated that theta activity was increased at the point of maximal conflict in temporarily ambiguous sentences (i.e., the disambiguation word) compared with unambiguous sentences. In addition, N400 effects of ambiguity were observed in Experiment 1 during the post-disambiguation period, suggesting that semantic processing was facilitated for words in the unambiguous condition compared to the ambiguous condition. In Experiment 2, we focused on these same EEG/ERP markers and examined adaptive control effects during comprehension by comparing temporarily ambiguous sentences following unambiguous sentences (Unambiguous-Ambiguous condition) to those following temporarily ambiguous sentences (Ambiguous-Ambiguous condition).

### Experiment 2: methods

#### Participants

This experiment received research ethics committee approval from the UC Santa Cruz institutional review board. We used the *mlmpower* package (Enders et al., 2023) in *R* (R Core Team, 2013) to determine that a sample size of 40 participants and 35 trials per condition would enable us to detect a medium effect size (*R*^*2*^ = .13) for fixed effects using the multilevel modeling approach described below, based on α = 0.05 and (1-β) = 0.85. Forty participants were recruited from the University of California, Santa Cruz (23 females; 8 males; 0 nonbinary; 7 did not report; average age 19 [range 18–23] years). All participants were right-handed, native speakers of English. Owing to a recording error, data from one participant were not usable. In addition, data from one participant were excluded due to poor data quality (greater than 50% of trials were not usable). Thus, the final sample consisted of 38 participants. 

#### Task

The task was adapted from the language task used in Experiment 1 and presented by using Neurobehavioral Systems Presentation (www.neurobs.com). Specifically, the single-conflict condition (Unambiguous-Ambiguous) was created by pairing a filler sentence containing no lexically ambiguous words with a sentence containing a lexically ambiguous word, while the double-conflict condition (Ambiguous-Ambiguous) paired two sentences containing lexical ambiguity. Filler sentences containing no lexically ambiguous words followed each sentence pair in both conditions. The 105 experimental sentence sets used in Experiment 1 were distributed across six counterbalanced lists such that, across lists, all sentences appeared in one of the possible ambiguous sentence positions (i.e., in the second sentence position in the UA condition, the first sentence position in the AA condition, and the second sentence position in the AA condition). Each participant was presented with one of these lists. Thus, each participant read 35 items in each condition.

Comprehension questions appeared in their entirety after the filler sentence at the end of every trial set (trial set: experimental sentence pair + filler sentence). Questions referenced the preceding filler sentence. Comprehension questions were included in order to encourage attentive reading, and appeared filler sentences only, (average accuracy 96.7%; range 83–100%).

#### EEG recording and preprocessing

EEG recording and preprocessing was conducted as described in Experiment 1. On average, 10.08% of trials were rejected due to the presence of artifacts, with an average of 31 trials per condition remaining in each dataset (range 17–35).

#### Data analysis

Single-trial estimates of theta power at the disambiguation word, and N400 amplitude at the post-disambiguation word were computed as described in Experiment 1.

### Experiment 2: results

All statistical analyses were performed in *R* (R Core Team, 2013) using the *lme4* package (Bates et al., 2014). As in Experiment 1, significant effects of condition are reported below in the main text. Significant main effects of scalp distribution are not reported in the main text, because these are not interpretable other than to indicate that overall voltage values varied across electrodes. However, significant interactions of scalp distribution variables and condition are interpretable and are reported in the main text below. Please see supplemental materials for full model output.

#### Conflict-related theta activity at disambiguation word

We first assessed the extent to which condition predicted theta-activity in response to the disambiguation word on a trial-by-trial basis. We used a linear mixed effects regression model with fixed effects of condition (UA, AA) and scalp-distribution variables corresponding to X (front-to-back), Y (left-to-right), and Z (superior to inferior) coordinates for each electrode (Boudewyn et al., 2024; Winsler et al., 2018, 2023). Language task condition was treatment-coded using UA as the reference condition. The model intercept therefore reflects the reference level of condition (UA). Because we did not expect potential EEG effects would necessarily be present or equivalent at all electrode locations, we also included interaction terms between the X-Y-Z coordinates and condition to capture the scalp distribution of any observed effects of condition. For example, an interaction between condition and the X-distribution predictor would capture a difference between conditions that was maximal at anterior electrode sites. The random effects structure for the model included random intercepts for participants and items, and by-participant random slopes for the effect of condition.

**Fig. 5:**
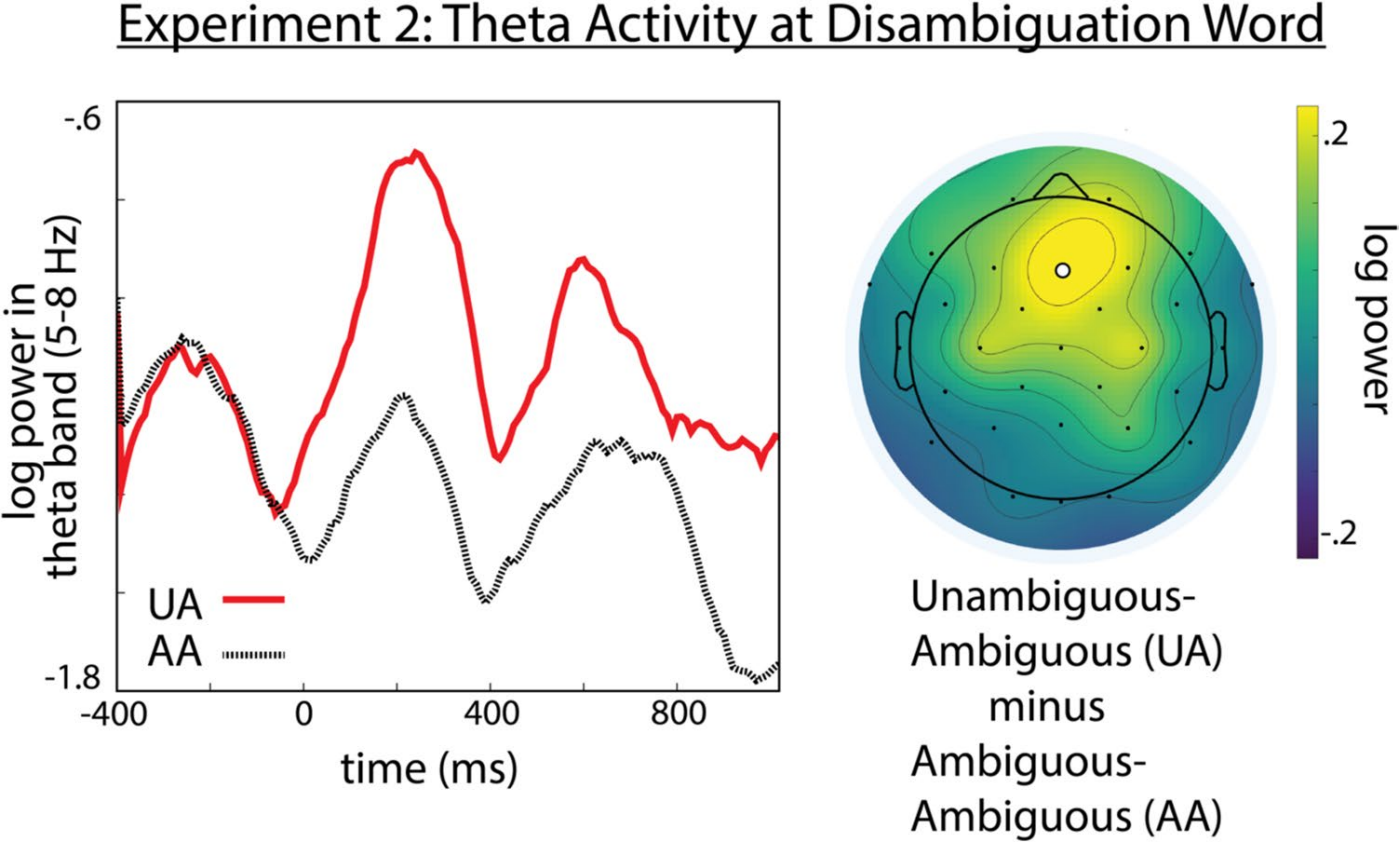
Conflict-related theta activity at disambiguation word in experiment 2. Left: Theta (5–8 Hz) power time-course time-locked to the disambiguation word at electrode Fz. Right: topographic map showing distribution of theta power for Ambiguous minus Unambiguous condition. Electrode Fz denoted by a white circle

The results showed a significant interaction between condition and the Y-distribution variable (*β* = −2.529e-04, *SE* = 1.275e-04, *p* = 0.0474). This interaction reflects a significant reduction in theta power for the AA compared with the UA condition at the disambiguation word, with this effect being maximal at right electrode sites. Results are summarized in Fig. 5.

#### Downstream effects on lexical semantic processing

We next assessed the extent to which language task condition predicted N400 amplitude to the post-disambiguation word on a trial-by-trial basis, using the same approach that we adopted to assess conflict-related theta activity at the disambiguation word. We fit a linear mixed-effects regression model including fixed effects of language task condition (UA, AA) and scalp-distribution variables corresponding to X (front-to-back), Y (left-to-right), and Z (superior to inferior) coordinates for each electrode, and interaction terms between condition and each of the X-Y-Z distribution predictors to capture the scalp distribution of any observed effects of condition. As above, language task condition was treatment-coded using UA as the reference condition. The model intercept therefore reflects the reference level of condition (UA). The random effects structure for the model included random intercepts for participants and items, and by-participant random slopes for the effect of condition.

**Fig. 6:**
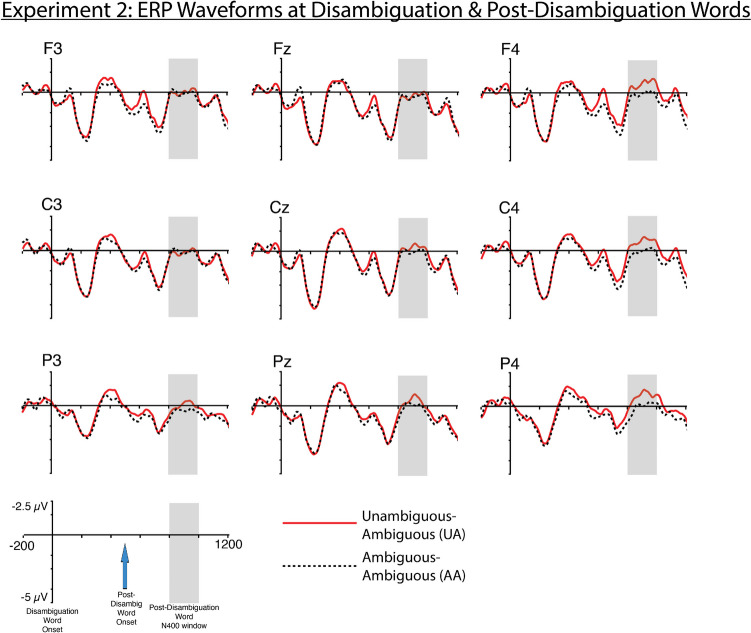
Experiment 2 ERP waveforms at the disambiguation and post-disambiguation words. Waveforms were time-locked to the disambiguation word in order to provide a visualization of ERP amplitudes throughout the disambiguation region. The post-disambiguation word onset 500 ms after the disambiguation word. Analysis focused on the N400 to the post-disambiguation word (shown in gray shading). Negative is plotted up

The results showed a significant interaction between condition and the X-distribution (*β* = 4.647e-03 ; *SE* = 2.14e-03; *p* = 0.0299) and Y-distribution (*β* = −4.234e-03; *SE* = 1.917e-03; *p* = 0.0272) parameters. These interactions reflect the significantly greater N400 amplitudes elicited by the UA condition at the post-disambiguation word at right posterior electrode sites compared with the AA condition (Fig. 6).

## General discussion

This study was designed to test the hypothesis that a common source of representational conflict during language comprehension (lexical ambiguity) engages domain-general neural computations associated with cognitive control, namely theta oscillations. We conducted two experiments to test this hypothesis. In Experiment 1, we examined the neural signature elicited by lexically ambiguous compared to unambiguous words during sentence comprehension. In Experiment 2, we looked for the presence of postconflict adaptation effects during language comprehension by comparing temporarily ambiguous sentences that followed a previous instance of ambiguity (conflict) to those that followed a previous low conflict sentence. We discuss our findings below.

### Linguistic conflict detection during language comprehension

Experiment 1 used EEG to compare the processing of unambiguous sentences to sentences that were temporarily ambiguous, in which the ambiguity was ultimately resolved in favor of the subordinate meaning of a lexically ambiguous word (e.g., *The ball/party was by invitation only*). The results of Experiment 1 showed a robust increase in mid-frontal theta oscillations in response to the disambiguation word (point of maximal conflict) in the ambiguous compared to unambiguous condition. We also hypothesized that once engaged by linguistic conflict, the cognitive control response would confer processing benefits on subsequently encountered sentences that also contained lexically ambiguous words, akin to the adaptive control or congruency sequence effects that have been observed outside of the language domain (Gratton et al., 1992; Ullsperger et al., 2005). Experiment 2 tested this by comparing the processing of temporarily ambiguous sentence trials that followed either a previous temporarily ambiguous sentence trial or an unambiguous sentence trial. As in Experiment 1, mid-frontal theta activity in Experiment 2 was observed at the disambiguation word (point of maximal representational conflict) of temporarily ambiguous sentences. Consistent with our hypothesis, this conflict-related mid-frontal theta response was reduced following previous conflict trials (AA condition) compared with nonconflict trials (UA condition). This trial-to-trial adaptive control effect observed in Experiment 2 extends previous behavioral work that has found postconflict facilitation effects on reaction times and/or accuracy within the context of a single-domain task (Akçay & Hazeltine, 2011; Egner, 2007; Egner et al., 2007; Kim et al., 2012; Notebaert & Verguts, 2008; Verguts & Notebaert, 2008, 2009). Previous EEG work that has found similar mid-frontal theta adaptive control effects with cognitive control tasks (Boudewyn & Carter, 2018; Valadez & Simons, 2018). It is also consistent with recent work in the language domain that has used cross-task adaptive control paradigms in which cognitive control task trials are interleaved with language comprehension trials, finding that the postconflict processing benefit generalized to linguistic conflict trials following high-conflict cognitive control task trials (Hsu et al., 2021; Hsu & Novick, 2016; Ovans et al., 2022; Thothathiri et al., 2018).

The theta effects elicited by linguistic conflict in both Experiment 1 and 2 were consistent in both their timing and scalp distribution to conflict-related theta activity that has been observed in a variety of cognitive control tasks outside of the language domain (Boudewyn & Carter, 2018; Cavanagh et al., 2012; Cavanagh & Frank, 2014; Cohen & Donner, 2013; Eschmann et al., 2018; Nigbur et al., 2012; Oehrn et al., 2014). Theoretically, midfrontal theta is generated by anterior cingulate cortex and signals the need for increased cognitive control to help adjudicate among multiple representations that simultaneously active (Botvinick et al., 2001, 2004; Cavanagh et al., 2012; Cavanagh & Frank, 2014; Kerns et al., 2004). Extending this set of models to language comprehension, Ness et al. (2023) propose that the same type of conflict detection process serves as a signal to engage a top-down biasing mechanism to strengthen the activation of the most likely of all active linguistic knowledge representations during comprehension. However, as noted in the introduction, to our knowledge, it is has not yet been demonstrated that a theta response analogous to that observed in non-linguistic cognitive control tasks is elicited by the presence of linguistic conflict during comprehension. We suggest that the midfrontal theta effects we observed in the current study in response to the disambiguation words in both experiments demonstrates that domain general neural computations are engaged by encountering a common source of linguistic conflict during comprehension (lexical ambiguity).

### Postconflict lexical semantic processing

In both Experiment 1 and Experiment 2, we sought to examine lexical semantic processing in the post-disambiguation period, after conflict detection has occurred. In Experiment 1, N400 amplitudes at the post-disambiguation word varied as a function of ambiguity condition: the N400 was reduced following an unambiguous word compared with following an ambiguous word. Note that this comparison is for the very same words; sentences only varied across condition in the ambiguous/unambiguous word that preceded this comparison point, with item sets being counterbalanced across participants. This pattern likely reflects facilitated lexical semantic processing for the unambiguous related to ambiguous condition, and is consistent with a similar pattern reported by Hagoort & Brown (2015).

We interpret this effect as evidence that lexical semantic processing is easier when multiple competing meaning representations are not in play, because the N400 was reduced in the unambiguous condition. There is a rich literature showing that the N400 not only serves as a marker of the relative ease of lexical semantic processing, but that it is very closely tied to how well a word fits with preceding context: N400 amplitudes reduce as predictability given prior context increases (Brothers et al., 2015; Brouwer et al., 2017; Federmeier & Kutas, 1999; Kutas & Hillyard, 1980; Rabovsky, 2020; Van Berkum et al., 1999, 2005). Recent computational modeling work has hypothesized that the N400 can be characterized as a marker of the amount of prediction error generated during word processing in context (Eddine et al., 2022, 2024). We suggest that under conditions of linguistic ambiguity, the expectations that can be generated about upcoming words are less certain, because there are multiple possible interpretations that are compatible with the input. Thus, identical words that appear in very similar sentences with very similar sentence-level meanings (i.e., *The ball/party was by invitation only*) would be expected to receive less of a processing benefit from context when previous context is ambiguous compared with when it was not. The current results support this hypothesis and support the idea that predictions about upcoming input are at their strongest in unambiguous context.

In Experiment 2, we again examined N400 amplitudes in response to the post-disambiguation word. We observed significantly reduced N400 amplitudes to post-disambiguation words in the AA condition compared with UA condition. This pattern of results demonstrates that the presence of lexical ambiguity on a particular trial (and presumably, the conflict detection signal that it elicited) ultimately led to facilitated lexical-semantic processing on the subsequent trial. Note that the comparison here was between two conditions in which previous ambiguity likely led to a higher degree of prediction error for incoming words, as established in Experiment 1. Therefore, Experiment 2 shows that even under ambiguous conditions in which very strong predictions about upcoming input are not possible, some degree of facilitation on lexical semantic processing is achieved by having recently encountered another instance of linguistic ambiguity.

This could be interpreted as the result of changes in expectancies about upcoming input, such that recent experience with a linguistic conflict trial changed expectations about the subsequent trial, boosting the activation level of the subordinate meaning of the lexically ambiguous word and ultimately leading to facilitated lexical semantic processing in the post-disambiguation period. However, given the midfrontal theta effects at the disambiguation word, which suggest that conflict-control processes were facilitated by repeated instances of linguistic conflict, we suggest that the N400 effects at the post-disambiguation word in Experiment 2 can be at least partly attributed to a cognitive control effect. Namely, we suggest that the repeated instances of linguistic conflict benefited from an already-engaged conflict-control mechanism, leading to the N400 effect when comparing UA to AA conditions.

## Conclusions

Overall, the results of this study showed that lexical ambiguity, a common source of representational conflict during language comprehension, elicits domain-general conflict-related theta activity. We also observed facilitatory effects on lexical semantic processing after the point of conflict. These results add to the growing literature that posits a role for domain general cognitive control in language comprehension (Boudewyn et al., 2012b, 2012a; Brothers et al., 2022; Hsu et al., 2021; Hsu & Novick, 2016; Ness et al., 2023; Nozari & Novick, 2017; Ovans et al., 2022; Thothathiri et al., 2018) and specifically suggest that the same neural computations are involved in processing nonlinguistic and linguistic conflict.

## Supplementary Information

Below is the link to the electronic supplementary material.

Supplementary file1 (DOCX 17 KB)

## Data Availability

The data reported here is available through the Open Science Framework: https://osf.io/64kev/.

## Code availability

Key scripts used in analyzing the data reported here are available through the Open Science Framework: https://osf.io/64kev/.
