# Supplementary material for: Common sources of linguistic conflict engage domain-general conflict control mechanisms during language comprehension
Source: Cogn Affect Behav Neurosci. 2025 Feb 12;25(3):744–56. doi: 10.3758/s13415-025-01267-3 (PMC12130101; doi:10.3758/s13415-025-01267-3)
Supplement: Supplementary file 1 — Supplementary file1 (DOCX 17 KB) [file 13415_2025_1267_MOESM1_ESM.docx]

Supplemental Materials

Experiment 1

*Full Model Output: Conflict-Related Theta Activity at Disambiguation Word*

****p<0.001 *p<0.05 ^p<0.07*

| Parameter | β | SE | p |
| --- | --- | --- | --- |
| Condition | -2.271e-02 | 1.886e-02 | 0.2373 |
| X | 6.014e-04 | 9.166e-05 | 5.35e-11*** |
| Y | -3.466e-04 | 8.212e-05 | 2.44e-05*** |
| Z | 5.740e-04 | 1.281e-04 | 7.41e-06*** |
| Condition x X | -3.304e-04 | 1.297e-04 | 0.0109* |
| Condition x Y | 2.273e-04 | 1.162e-04 | 0.0505^ |
| Condition x Z | -4.262e-04 | 1.813e-04 | 0.0187* |

Experiment 1

*Full Model Output: Downstream Effects on Lexical Semantic Processing*

****p<0.001 *p<0.05 ^p<0.07*

| Parameter | β | SE | p |
| --- | --- | --- | --- |
| Condition | -8.051e-02 | 4.282e-01 | 0.0947 |
| X | 5.046e-03 | 1.255e-03 | 5.84e-05*** |
| Y | 1.664e-05 | 1.124e-03 | 0.9882 |
| Z | 8.063e-03 | 1.753e-03 | 4.25e-06*** |
| Condition x X | 1.658e-03 | 1.778e-03 | 0.3510 |
| Condition x Y | 2.112e-03 | 1.592e-03 | 0.1847 |
| Condition x Z | -5.181e-03 | 2.483e-03 | 0.0369* |

Experiment 2

*Full Model Output: Conflict-Related Theta Activity at Disambiguation Word*

****p<0.001 *p<0.05 ^p<0.07*

| Parameter | β | SE | p |
| --- | --- | --- | --- |
| Condition | -5.044e-03 | 1.812e-02 | 0.7821 |
| X | 6.057e-04 | 1.008e-04 | 1.85e-09*** |
| Y | 1.383e-04 | 9.028e-05 | 0.1256 |
| Z | 9.997e-05 | 1.408e-04 | 0.4777 |
| Condition x X | 1.834e-04 | 1.423e-04 | 0.1975 |
| Condition x Y | -2.529e-04 | 1.275e-04 | 0.0474* |
| Condition x Z | 1.703e-04 | 1.989e-04 | 0.3920 |

Experiment 2

*Full Model Output: Downstream Effects on Lexical Semantic Processing*

****p<0.001 *p<0.05 ^p<0.07*

| Parameter | β | SE | p |
| --- | --- | --- | --- |
| Condition | 2.637e-01 | 3.816e-01 | 0.4936 |
| X | -1.739e-03 | 1.515e-03 | 0.2511 |
| Y | 5.779e-03 | 1.357e-03 | 2.06e-05*** |
| Z | 1.833e-03 | 2.117e-03 | 0.3864 |
| Condition x X | 4.647e-03 | 2.140e-03 | 0.0299* |
| Condition x Y | -4.234e-03 | 1.917e-03 | 0.0272* |
| Condition x Z | -3.507e-03 | 2.989e-03 | 0.2407 |
